# Late I_Na_ as a Therapeutic Target: New Strategies, Computational Modelling, Drug Development, and Clinical Translation

**DOI:** 10.1002/med.70077

**Published:** 2026-07-17

**Authors:** Arkapravo Chattopadhyay, Lynn C. Lunsonga, Peter E. Light, Khaled Barakat

**Affiliations:** ^1^ Faculty of Pharmacy and Pharmaceutical Sciences University of Alberta Edmonton Alberta Canada; ^2^ Faculty of Medicine and Dentistry, Department of Pharmacology University of Alberta Edmonton Alberta Canada

**Keywords:** late INa, multiscale modeling, Nav1.5, SGLT2 inhibitors, sodium channel mutations

## Abstract

The Nav1.5 channel, a major isoform of voltage‐gated sodium ion channel, is mainly found in ventricular cardiomyocytes, playing a key role in generating essential cardiac action potentials for normal heart rhythms. Mutations in Nav1.5 have been associated with severe heart conditions such as long QT syndrome, Brugada syndrome, cardiac conduction disorders, atrial fibrillation, and dilated cardiomyopathy. Recent research has linked Nav1.5 to cardiac fibrosis and proposed its role in non‐cardiac illnesses, including specific neurological disorders and cancers, subjects that will be reviewed in this paper. On the other hand, sodium‐glucose cotransporter 2 inhibitors (SGLT2i), initially designed to manage diabetes by facilitating glucose excretion through urine, have demonstrated unexpected and encouraging cardioprotective benefits in clinical trials. This review compares the important SGLT2 inhibitors empagliflozin, dapagliflozin, and canagliflozin in terms of their interactions with Nav1.5 and their therapeutic effects on the heart. We also investigate new medications and compounds being developed to regulate Nav1.5 function, providing a preview of potential future treatments. Past attempts to develop late I_Na_ inhibitors and difficulties in transitioning from the research phase to clinical trials have raised doubts about the optimal design of such trials. In addition, we cover the applications of molecular dynamics simulations in understanding the mechanism of action of these drugs within the Nav1.5 channel computationally. We hope that this review identifies new opportunities to generate more effective inhibitors using novel methods and advanced multiscale molecular modelling techniques.

## 1 Introduction

Voltage‐gated sodium channels (VGSCs) are vital for the rapid initiation and propagation of electrical signals in excitable tissues. Among the nine known isoforms, Nav1.5, which is predominantly expressed in ventricular cardiomyocytes, plays a central role in generating cardiac action potentials (CAP). This channel is crucial for maintaining normal heart rhythms, and disruptions in its function are associated with a broad spectrum of cardiac disorders. Numerous cardiac conditions are associated with Nav1.5 dysfunction. Notably, long QT syndrome type 3 (LQT3) has been associated with specific *SCN5A* gene mutations that increase late sodium current.

First, it is essential to understand the distinction between peak sodium ion current (peak I_Na_) and late sodium ion current (late I_Na_). Upon membrane depolarization, the voltage‐sensing domains (VSDs) of the Nav1.5 channel transform conformational changes that rapidly open the channel pore, allowing a transient influx of Na^+^ ions. This generates the peak I_Na_, responsible for the phase 0 upstroke of the CAP and ensures rapid excitation and conduction across cardiomyocytes [1]. Shortly after activation, the channel enters a fast‐inactivated state, mainly facilitated by VSDs in domains III and IV, effectively terminating peak I_Na_ to maintain electrical stability [2]. In contrast, a small persistent current, known as the late I_Na_, continues during the plateau phase (phase 2) of the CAP. Although it typically constitutes less than 0.5% of peak I_Na_ and serves a modest role in CAP under normal physiological conditions [3]. However, in pathological conditions, when the amount of late I_Na_ increases, it raises the risk of arrhythmia and prolongs action potentials [4]. However, not all Nav1.5 mutations affect late I_Na_. For instance, mutations that cause dilated cardiomyopathy or Brugada syndrome (BrS) more frequently result in altered channel trafficking or a loss of function in peak sodium current [5, 6]. Therefore, it is essential to distinguish between diseases involving late versus peak I_Na_, even though late I_Na_ plays a crucial role in arrhythmogenesis, which makes it a promising therapeutic strategy for cardiac arrhythmias. However, past attempts to develop selective inhibitors of late I_Na_ have faced significant challenges, including off‐target effects, suboptimal pharmacokinetics, and limited clinical efficacy [7, 8, 9]. The complex role of late I_Na_ in both physiological and pathological states further complicates the design of safe and effective inhibitors.

Interestingly, sodium‐glucose cotransporter 2 (SGLT2) inhibitors, initially developed for managing type 2 diabetes, have demonstrated unexpected cardiovascular benefits [10]. Recent studies suggest that these agents selectively inhibit late I_Na_, offering a novel mechanism for their cardioprotective effects. Compounds like empagliflozin, dapagliflozin, and canagliflozin have shown promise in reducing cardiac arrhythmias, sparking interest in their therapeutic potential beyond glucose regulation [11, 12, 13]

Advancements in computational modeling have also opened new avenues for understanding Nav1.5′s structural and functional dynamics. Techniques such as molecular dynamics simulations and multiscale modeling have provided unprecedented insights into the channel's conformational states, drug‐binding interactions, and mutation‐induced dysfunctions [14, 15, 16, 17, 18, 19]. These methods not only accelerate drug discovery but also enhance our understanding of the biophysical principles underlying channel activity.

The therapeutic importance of Nav1.5 is further supported by the fact that it is expressed in cell types other than heart tissue and has been linked to non‐cardiac diseases, such as neurological illnesses and certain types of cancer [20].

This review focuses on the late sodium current (late I_Na_) as a therapeutic target in Nav1.5‐associated diseases. We examine the mechanistic basis of late I_Na_, its role in disease, and current and emerging strategies for selective modulation, including SGLT2 inhibitors and computational drug‐discovery approaches. By synthesizing recent advances in molecular biology, pharmacology, and computational modeling, this review seeks to identify actionable insights for the development of next‐generation therapies for Nav1.5‐associated diseases.

## 2 Nav1.5: Genetic Regulation, Structure, and Role in Cardiac Electrophysiology

### 2.1 Nav1.5 Genetic Regulation

*SCN5A*, or VGSC, type V alpha subunit gene (ENSG00000183873, HGNC:10593, NCBI Gene ID: 6331), belongs to a family of ten genes that encode sodium channel alpha subunits. Nav1.5, encoded by *SCN5A* gene, situated on chromosome 3p22 within the human genome and consisting of 28 exons, is the main sodium channel in the heart. Veerman et al., [21]. Although multiple sodium channel isoforms exist across excitable tissues, Nav1.5 plays a dominant role in cardiac depolarization and conduction.

Comprising 2016 amino acids [1], the human Nav1.5 protein is composed of a single α subunit and four β subunits, where the β subunits play crucial roles in regulating and localizing the channel within the cellular environment. Veerman et al., [21]. Mutations in SCN5A are strongly linked to inherited and acquired arrhythmias. From a therapeutic perspective, these mutations can differentially affect peak I_Na_ or late I_Na_, with gain‐of‐function alterations in channel inactivation often leading to enhanced late I_Na_ and increased arrhythmogenic risk.

### 2.2 Structure and Critical Residues

Nav1.5 represents a VGSC, comprised of a singular pore‐forming α subunit featuring four structurally homologous but nonidentical transmembrane domains (DI–DIV) (DI, aa 127–416; DII, aa 712–939; DIII, aa 1201–1470; D4, aa 1524–1772) [1, 22]. The pore region, defined by P‐loops between S5 and S6, contains key selectivity filters (EEDD ring ‐ E375 (DI), E901 (DII), D1423 (DIII), and D1714 (DIV), and DEKA ring‐ D372 (DI), E898 (DII), K1419 (DIII), and A1711 (DIV) rings) that govern sodium permeation. Ahmed et al., [18]. Mutations within these residues could alter the selectivity property of hNav1.5 [23].

In the ID1‐2 linker, Serine 571 is a vital phosphorylation site for calcium/calmodulin‐dependent protein kinase II. The IFMT motif (isoleucine‐phenylalanine‐methionine‐threonine, 1485–1488) in the linker of ID3‐4 is responsible for rapid channel inactivation. Recent structural insights have further refined this mechanism by identifying residue‐level interactions that stabilize the inactivated state. Key contributors include residues from DIII (E1318, A1326, and G1329) and DIV (N1659), which interact with the IFMT motif and with neighboring residues N1472 and Q1476 within the DIII–DIV linker. These interactions are mediated primarily by hydrogen bonds, forming specific residue pairs such as N1472–G1329, Q1476–A1326, F1486–N1659, and T1488–E1318 [2, 19]. Collectively, these interactions constitute key hydrogen bond pairs that stabilize the inactivated conformation. From a therapeutic point of view, these residue‐level insights are especially useful because they indicate that selectively modulating inactivation and stabilizing interactions, rather than employing broad channel blockade, could be an effective approach for targeting late I_Na_ while maintaining normal peak I_Na_. Figure 1 illustrates the structure of hNav1.5 in detail.

**Figure 1:**
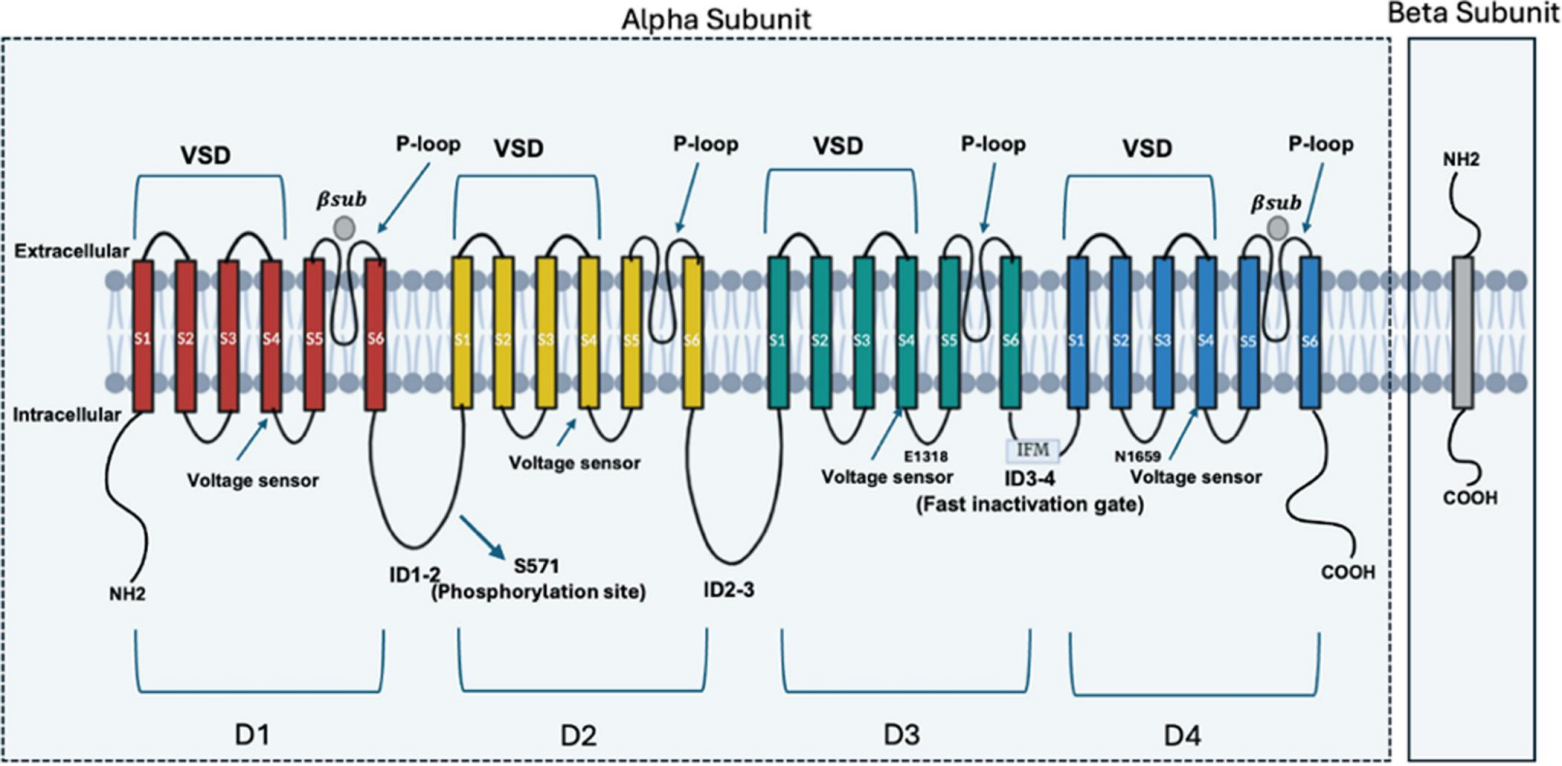
Structure of human voltage‐gated sodium Channel 1.5. Domains (D1–D4) of the alpha subunit of Nav1.5 are in red, yellow, green, and blue colors, respectively; IFMT motifs; and beta subunits are in gray. [Color figure can be viewed at wileyonlinelibrary.com]

### 2.3 Functional Role of Nav1.5 in Cardiac Electrophysiology

Nav1.5 mediates the rapid upstroke (phase 0) of the CAP via peak I_Na_, enabling rapid electrical conduction across cardiomyocytes. After activation, most channels rapidly transition to an inactivated state; however, a small fraction may remain open or reopen, giving rise to late I_Na_ during the plateau phase [24]. Although late I_Na_ is a minor component of the total sodium current under physiological conditions, its contribution becomes significant in disease states. Even modest increases in this sustained inward current can prolong action potential duration, disrupt ionic balance, and promote arrhythmogenic events, including early afterdepolarizations [25]. From a therapeutic perspective, this uneven effect makes late I_Na_ an appealing target, since selective inhibition could lower arrhythmia risk while maintaining the vital role of peak INa in conduction. Figure 2 depicts all the steps involved in CAP.

**Figure 2:**
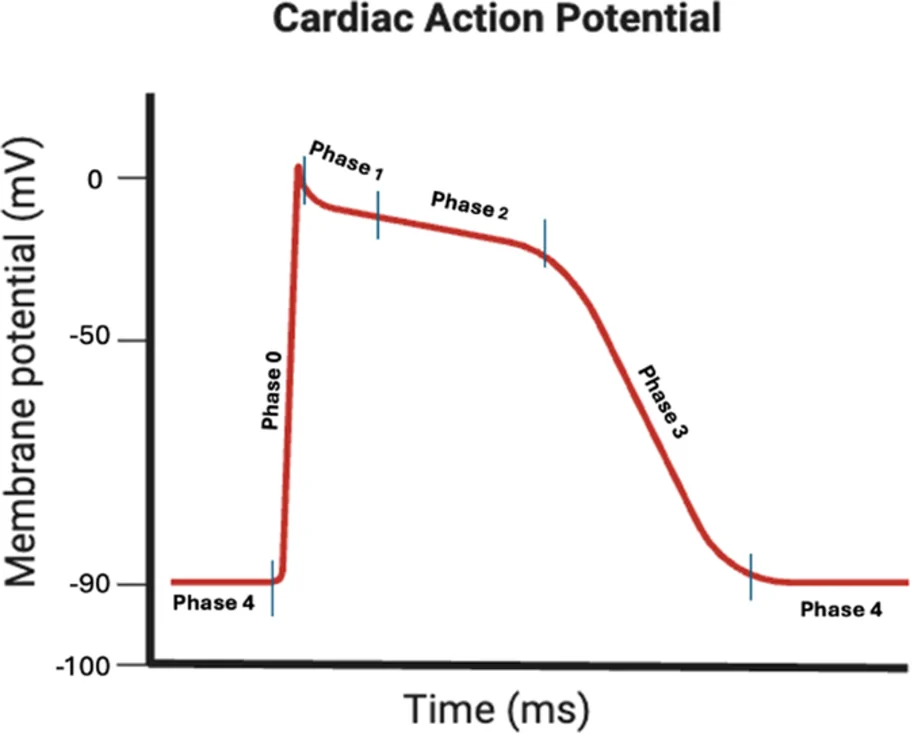
Action potential of cardiac muscle cell shown in different phase w.r.t. time and membrane potential (mV). [Color figure can be viewed at wileyonlinelibrary.com]

### 2.4 Fast Inactivation as a Key Determinant of Late I_Na_

Fast inactivation is a vital control process that restricts sodium entry after channel activation. It is mediated by the DIII–DIV linker, functioning as a “hinged lid” that blocks the pore soon after it opens. The IFMT motif plays a key role in this process, and mutations like IFM → QQQ that disrupt this region eliminate inactivation, leading to a continuous sodium current [26]. Impairment of fast inactivation is one mechanism behind pathological late I_Na_. Nonetheless, late I_Na_ can also result from other processes, such as changes in voltage sensor behavior, post‐translational modifications like phosphorylation, and oxidative stress. This variety of mechanisms complicates drug development, as various pathological situations might need different approaches to effectively suppress late I_Na_ [27]. Overall, these findings highlight that effectively targeting late I_Na_ involves not just inhibiting the persistent current but also understanding the structural and regulatory mechanisms that produce it. These structural features are directly related to late I_Na_ because disruptions in inactivation gating and channel conformational dynamics play a crucial role in determining the persistent sodium current.

## 3 Regulation of Late I_Na_: Role of Phosphorylation and Post‐Translational Modifications

### 3.1 Biophysical Distinction Between Late I_Na_ and Peak I_Na_

As mentioned earlier, peak I_Na_ refers to the rapid, transient sodium influx that drives the action potential upstroke, while late I_Na_ is a smaller, sustained current resulting from incomplete channel inactivation. Despite its minor size, late I_Na_ significantly affects action potential duration and the development of arrhythmias. This difference is clinically significant because selectively blocking late I_Na_ could decrease arrhythmic risk without impairing peak I_Na_ and normal electrical conduction [2]. Although late I_Na_ is small in amplitude, it significantly influences the duration of the action potential and arrhythmogenesis.

From a therapeutic standpoint, this functional separation is essential. Selectively inhibiting late I_Na_ could normalize repolarization and lower arrhythmic risk while preserving peak I_Na_, thereby maintaining normal conduction. Nonetheless, achieving such selectivity is difficult because both currents originate from the same channel and are controlled by overlapping structural mechanisms and determinants. The temporal, biophysical, and pathological distinctions between peak and late I_Na_ in both healthy and diseased states are depicted in Figure 3.

**Figure 3:**
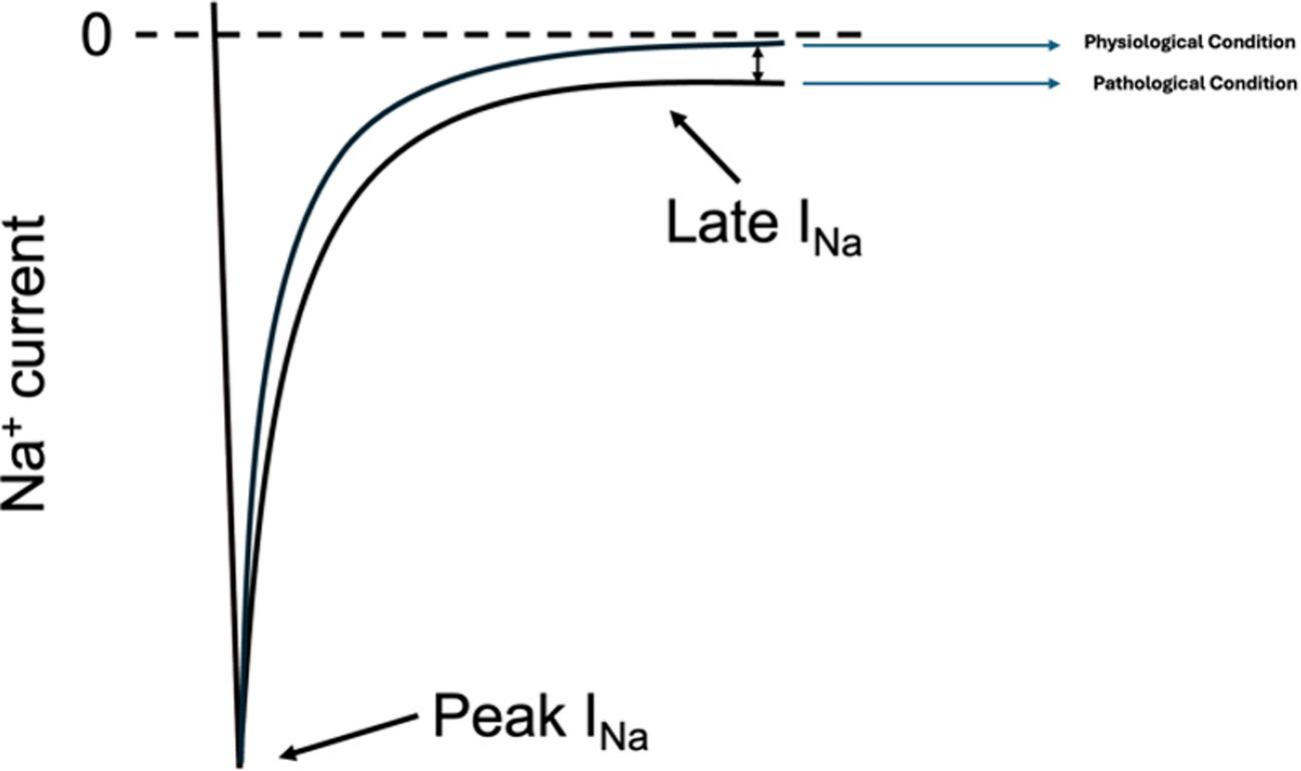
Late and peak I_Na_ in physiologic and pathological conditions. [Color figure can be viewed at wileyonlinelibrary.com]

### 3.2 Different Pathways of Phosphorylation and Its Effect on Late I_Na_

Two pathways have been identified that enhance late I_Na_ by modifying Scn5a through post‐translational processes. One pathway involves CaMKII‐dependent phosphorylation, whereas the other pathway involves neuronal nitric oxide synthase (nNOS) dependent nitrosylation.

1. CaMKII‐dependent phosphorylation: One well‐defined pathway that increases late I_Na_ involves CaMKII‐dependent phosphorylation of Nav1.5. The leading key phosphorylation site is S571, situated in the loop between DI and DII, although S516 and T594 are important phosphorylation sites. CaMKII modulates the gating properties of Nav1.5 and increases the late sodium current, playing a crucial role in regulating cardiac sodium channels. Recent research has focused on the role of Nav1.5 as a target for regulation by intracellular calcium (Ca2+), calmodulin (CaM), and Ca2+/calmodulin‐dependent protein kinase II (CaMKII). In humans, Nav1.5 interacts with CaM through specific regions, including an IQ motif in the C‐terminal tail and a segment in the DIII‐DIV linker (1520‐FIF‐1522 and 1494‐YY‐1495) [27, 28, 29]. However, the functional consequences of this interaction are inconsistent, affecting parameters such as steady‐state inactivation and voltage dependence of activation in different ways. It is important to note that although CaM is traditionally known as a Ca2+‐binding protein, there is evidence of direct binding between calcium ions and Nav1.5, which could potentially link Ca2+ binding to CaM interaction. In addition, CaMKII also plays a role in modulating Nav1.5, influencing characteristics of sodium channel gating and increasing late I_Na_ [30].
2. nNOS‐dependent nitrosylation: Another pathway involves nNOS‐dependent S‐nitrosylation of Nav1.5. This process has been shown to elevate late I_Na_, though the specific cysteine residue(s) involved are still unknown. Ueda et al., [31] nNOS functions within a macromolecular complex that includes SNTA1 and PMCA4b, which together regulate nitric oxide signaling at the channel. Disruption of this complex—such as through mutations like A390V in SNTA1—reduces inhibition of nNOS, resulting in increased nitrosylation of Nav1.5 and an increase in late I_Na_. This mechanism is linked to LQT3, highlighting the importance of post‐translational modifications in arrhythmia development [32].

### 3.3 Critical Considerations and Therapeutic Implications

Although CaMKII phosphorylation and nNOS‐mediated nitrosylation are often studied separately, their potential interaction remains poorly understood. In addition, variations across experimental studies due to differences in model systems, channel isoforms, and electrophysiological protocols hinder direct comparison and clinical translation [31].

From a therapeutic perspective, these findings present both opportunities and challenges. Targeting upstream regulators like CaMKII or nNOS might offer indirect methods to reduce late I_Na_, but such strategies could cause off‐target effects because of their broad physiological roles. Alternatively, directly stabilizing channel inactivation, perhaps by modulating structural elements like the inactivation gate or interaction networks, may achieve greater specificity. Overall, this evidence suggests that late I_Na_ results from a complex interaction of structural and regulatory components, making a deeper mechanistic understanding essential for developing targeted, selective therapies.

## 4 Nav1.5 in Cardiac and Non‐Cardiac Disease: Relevance to Late I_Na_

Nav1.5 is mainly found in heart tissue, where it controls electrical signaling. However, it has also been observed in non‐cardiac systems such as neuronal, gastrointestinal, and cancer cells. Although these additional roles are gaining recognition, their significance to late I_Na_ and therapeutic targeting varies considerably. Wang et al., [33] Therefore, this section mainly discusses disease contexts where late I_Na_ has a confirmed or likely pathophysiological role.

### 4.1 Cardiac Diseases

#### 4.1.1 Arrhythmias and Mutation‐Specific Effects

Today, cardiovascular disease is globally among the top causes of mortality [34]. When Nav1.5 is impaired, it can lead to various cardiac arrhythmias, which can be inherited or acquired and are often associated with different cardiovascular diseases [35]. Nav1.5 dysfunction is closely linked to cardiac arrhythmias, caused by either gain‐ or loss‐of‐function mutations in the *SCN5A* gene. These mutations lead to different electrophysiological effects: loss‐of‐function variants usually decrease peak I_Na_ and slow conduction, while gain‐of‐function variants often increase late I_Na_ by destabilizing channel inactivation [36].

Another disorder involving *SCN5A* gene mutations is catecholaminergic polymorphic ventricular tachycardia, which is characterized by stress‐induced atrial and ventricular arrhythmias that result in abrupt cardiac death. Mutations like L807P and E446K raise late I_Na_ and destabilize Nav1.5 inactivation [37]. This distinction is important because only some Nav1.5‐related disorders are caused by elevated late I_Na_. As a result, treatments that focus on late I_Na_ are likely to be most beneficial in cases with ongoing sodium influx, rather than in those mainly involving slower conduction.

#### 4.1.2 Long QT Spagesyndrome (LQTS)

LQT3 is a well‐known disease model of pathological late I_Na_, caused by gain‐of‐function mutations in Nav1.5. These mutations impair fast inactivation, leading to a sustained inward sodium current that extends the duration of the action potential [38, 39]. Specifically, gain‐of‐function Nav1.5 mutations that increase the late I_Na_ are the cause of LQT3. Repolarization is delayed by this continuous current, which lengthens the CAP plateau phase [40, 41]. EADs are encouraged by elevated late I_Na_, which also interferes with calcium homeostasis through the Na+/Ca2+ exchanger. LQT3 symptoms typically appear during rest or sleep, in contrast to LQT1 and LQT2, which are associated with potassium channel failure [42, 43, 44]. The strong mechanistic link between late I_Na_ and disease pathology in LQT3 supports targeting late INa as a rational therapeutic strategy.

#### 4.1.3 Brugada Syndrome (BrS)

In contrast, BrS is mainly linked to loss‐of‐function mutations in nav1.5 that diminish peak I_Na_, resulting in impaired depolarization and conduction issues [45, 46]. Although BrS shares a genetic connection with LQT3, its core mechanism is mostly separate from late I_Na_ [47, 48]. This distinction emphasizes a key therapeutic limitation. Methods focused on suppressing late I_Na_ probably won't be effective for conditions caused by decreased peak current. Therefore, not all Nav1.5‐related arrhythmias are appropriate for late I_Na_ modulation.

#### 4.1.4 Other Cardiac Diseases

Nav1.5 dysfunction has been linked to cardiomyopathies and metabolic disorders, with changes in channel trafficking, phosphorylation, or gating kinetics potentially playing a role in disease development. However, in many cases, the role of late I_Na_ is less well‐defined or appears to be secondary to other mechanisms [49]. This highlights an important point. Although late I_Na_ is a significant factor in arrhythmogenesis, it should not be considered the cause of all Nav1.5‐related conditions. Therefore, thorough mechanistic differentiation is crucial to inform therapeutic strategies.

### 4.2 Non‐Cardiac Diseases

#### 4.2.1 Cancer

Nav1.5 is abnormally expressed in various cancers, such as breast, prostate, and colon carcinomas, playing a role in promoting invasion and metastasis instead of electrical activity. Importantly, cancer cells frequently produce a neonatal splice variant (nNav1.5) that has unique biophysical characteristics [50]. Functionally, ongoing sodium influx, including from late I_Na_, is associated with higher intracellular sodium, activation of the Na^+^/H^+^ exchanger (NHE1), and extracellular acidification, all of which facilitate tumor invasion and metastasis. Notably, blocking late I_Na_ with drugs like ranolazine has been demonstrated to decrease metastatic potential in preclinical studies [51]. However, despite these promising findings, the therapeutic relevance of targeting late I_Na_ in cancer remains exploratory, and the absence of selective inhibitors, particularly for nNav1.5, represents a major limitation.

#### 4.2.2 Neurological Disorders

Nav1.5 is expressed in specific regions of the nervous system, including the cortex, hippocampus, and dorsal root ganglia, where it may play a role in neuronal excitability [52, 53, 54]. Emerging evidence associates nav1.5 variants with epilepsy, with certain gain‐of‐function mutations potentially increasing late I_Na_ and contributing to neuronal hyperexcitability [55, 56]. However, this relationship remains largely correlational, and the contribution of late I_Na_ has not been definitively established [57, 58]. Notably, the coexistence of cardiac arrhythmias and seizure phenotypes, along with associations with sudden unexpected death in epilepsy, indicates a possible shared mechanism [59, 60, 61]. Nonetheless, the role of late I_Na_ in neurological disorders remains not fully elucidated and warrants further investigation [62, 63, 64].

#### 4.2.3 Gastrointestinal Disorders

Nav1.5 has been associated with gastrointestinal motility disorders, including irritable bowel syndrome (IBS), with *SCN5A* gene variants identified in a small subset of patients (~2%) [65, 66]. Both gain‐ and loss‐of‐function mutations have been documented, impacting sodium channel activity to various extents. Although pharmacological modulation of Nav1.5 (e.g., mexiletine) has demonstrated symptomatic benefits in certain cases, the specific role of late I_Na_ remains indeterminate. Consequently, its significance as a therapeutic target in gastrointestinal diseases remains speculative [67].

## 5 Emerging Therapeutic Targets and Modulators of Nav1.5

Drugs or small molecules are usually known as common modulators for Nav1.5 but there are many non‐drug modulators, like enzymes or proteins, that directly or indirectly affect the action of Nav1.5. In this section, we will discuss different modulators that have a high impact on Nav1.5.

### 5.1 Nicotinamide Adenine Dinucleotide (NAD)

Nicotinamide riboside (NR), a promising modulator of cardiac sodium channel Nav1.5, has been identified in research on NAD+ supplementation. It can increase peak I_Na_ and decrease late I_Na_ in cardiomyocytes via both acetylation‐independent and acetylation‐dependent mechanisms. In animal models, NR supplementation demonstrated the ability to shorten the QTc interval in contrast to nicotinamide, which is consistent with its effects on lowering the late I_Na_. According to these results, NR supplementation may be a useful therapeutic approach for treating hereditary and acquired conditions associated with aberrant Na+ currents [68].

### 5.2 Capsaicin

A class of membrane‐active medications known as amphiphiles has been investigated by researchers as a possible therapeutic agent to minimize closed‐state inhibition of voltage‐gated currents while specifically targeting the mechanosensitive function of Nav1.5. Capsaicin was chosen by them for additional study among the evaluated amphiphiles. The study found that capsaicin effectively eliminated the mechanosensitive increases in peak current and altered the voltage dependence of activation and inactivation, using membrane suction in cell‐attached patches and fluid shear stress on entire cells. Furthermore, Capsaicin may be a targeted therapeutic intervention for disorders related to Nav1.5 mechanosensitive channels, as the study also found that mechanical stimuli may affect Nav1.5 mechanosensitive channels differently [69].

### 5.3 Protein 14‐3‐3

The impact of Protein‐protein interaction is enormous on the Nav1.5 protein. Zheng et al. [70] investigate how protein 14‐3‐3 regulates the heart sodium channel Nav1.5 in response to different antiarrhythmic medications (AADs), illuminating a new mechanism that may affect the effectiveness of treatment for cardiac arrhythmias. AADs are known to target Nav1.5, a protein essential for generating CAPs. However, the way these medications influence their activity may change if they are coupled with 14‐3‐3. Utilizing high‐throughput patch‐clamp tests on HEK293 cells that expressed Nav1.5, the researchers investigated the effects of flecainide, mexiletine, lidocaine, and quinidine under 14‐3‐3 inhibition. They found that blocking 14‐3‐3 increased the blocking action of quinidine, changed the dose and use‐dependency profiles of mexiletine, lidocaine, and quinidine, and caused Nav1.5 activation to shift toward hyperpolarized voltages for all four medications. Excitingly, it has also been found that when quinidine is present, the protein 14‐3‐3 inhibition also slowly initiates recovery from inactivation. This means that 14‐3‐3 affects the pharmacological profile of Nav1.5 by altering its biophysical characteristics in response to AADs, suggesting that interactions between 14‐3‐3 and Nav1.5 could be a useful factor to consider when developing next‐generation antiarrhythmic treatments.

### 5.4 p38 Mitogen‐Activated Protein Kinase (p38 MAPK)

p38 MAPK activation contributes to the age‐related decrease in Nav1.5 current density and the increased vulnerability to arrhythmias in aging hearts. Due to changes in gap junction protein Cx43, Nav1.5 function, and increased fibrosis, aging is linked to decreased atrial and ventricular conduction velocities, all of which increase the risk of arrhythmias. Here, researchers employed biochemical, electrophysiological, and cell imaging methods to demonstrate that the stress‐responsive kinase p38 MAPK becomes more active as mice age, resulting in cellular alterations that increase the risk of arrhythmias. In particular, in Nav1.5‐expressing HEK‐293T cells and human‐induced pluripotent stem cell‐derived cardiomyocytes (hiPSC‐CMs), p38 MAPK activation decreased both peak and late sodium current density and the maximum upstroke velocity (d_V_/dt_max_), but it did not affect the voltage dependency or total protein expression of Nav1.5. Notably, these decreases in sodium current and d_V_/dt_max_ were prevented by pre‐treating cells with a p38 MAPK inhibitor, indicating that p38 MAPK signaling plays a crucial role in age‐dependent Nav1.5 dysregulation. These results suggest that maintaining Nav1.5 function in the face of aging‐related cellular stress makes p38MAPK a promising therapeutic target for reducing the risk of arrhythmias in the elderly [71].

### 5.5 Dipeptidyl Peptidase‐Like Protein‐10 (DPP10)

It is a novel regulatory dipeptidyl peptidase‐like transmembrane protein in the human heart that has consequences for arrhythmogenesis, especially in heart failure. Nav1.5 channel complexes depend on cardiac auxiliary β‐subunits, which alter their biophysical characteristics and affect the risk of arrhythmias. In human ventricles, DPP10 was found to interact with Nav1.5 channels in addition to its well‐known regulation of Kv4.3 potassium channels. DPP10 expression was found to be increased in samples from heart failure patients. Action potential upstroke velocity was significantly reduced in rat cardiomyocytes with adenoviral DPP10 expression (DPP10ad), according to functional tests. This suggests that the Na+ current density has decreased. Furthermore, DPP10 increased the window current and the number of available channels for activation by shifting the voltage‐dependent activation and inactivation curves of Nav1.5 channels to more positive potentials. In addition, DPP10 promoted channel reopenings by decreasing the time‐to‐peak Na+ current and accelerating recovery from inactivation. Co‐immunoprecipitation tests in human ventricles verified the physical interactions between DPP10 and Nav1.5. According to these results, increased DPP10 expression in heart failure may increase the risk of arrhythmias by altering the function of Nav1.5 channels, which connect Na+ and K+ channel complexes, offering a novel therapeutic target [72].

### 5.6 POPDC Proteins

It was discovered that Popeye domain‐containing POPDC proteins, which are abundantly expressed in skeletal and cardiac muscle, have been demonstrated to affect the TREK‐1 potassium channel in a cAMP‐dependent way and also modulate Nav1.5. Through the use of functional screens in Xenopus oocytes, the researchers discovered that POPDC proteins reduce the surface expression of the channel, hence decreasing Nav1.5 currents in a cAMP‐dependent manner. Both Nav1.5 and POPDC2, which co‐immunoprecipitate with each other from cardiac tissues, are expressed in specific areas of the mouse heart, indicating that they interact physiologically. In addition, popdc2 knockdown in zebrafish resulted in increased upstroke velocity and CAP overshoot, indicating that decreased POPDC2 function increases Nav1.5 activity. In patients with POPDC1 or POPDC2 variants linked to conduction disorders, this modulation of Nav1.5 by POPDC proteins highlights a potential pathway influencing cardiac excitability and arrhythmogenic risk by introducing a novel mechanism by which cardiac sodium channel densities can be regulated under sympathetic stimulation [73].

### 5.7 Calmodulin (CaM)

CaM, a small, highly conserved calcium‐binding protein, regulates the ventricular sodium channel Nav1.5 [74]. CaM binding to Nav1.5 is essential for the heart channels to operate properly. BrS and LQT3 are two different cardiac arrhythmia syndromes caused by mutations in Nav1.5 that disrupt CaM binding. BrS is associated with a loss‐of‐function mutation that leads to decreased channel activity, whereas LQT3 is characterized by a gain‐of‐function mutation that causes prolonged action potentials. By using single‐channel analysis, this study demonstrates that when CaM binding to Nav1.5 is disrupted, the consequently altered inactivation dynamics lead to both an increase in persistent peak and late Na+ current and a decrease in channel activity. These results highlight a complex regulatory mechanism that, when weakened, predisposes people to severe arrhythmias with disparate pathophysiological profiles. They also clarify how distinct patterns of channel dysfunction arise from altered CaM‐NaV1.5 interactions.

In summary, in this section, we have learned about different non‐drug, enzyme, or protein modulators of Nav1.5 that have a significant impact on the VGSC. Next, we will go through the drug molecules that have immense direct or allosteric effects on Nav1.5.

## 6 History of Developing Classical and Emerging Nav1.5‐Targeting Drugs in Antiarrhythmic Therapy (Excluding SGLT2 Inhibitors)

Several small‐molecule drugs targeting Nav1.5 have been investigated for their therapeutic potential, particularly in the management of arrhythmias. First‐generation sodium channel inhibitors, including class I antiarrhythmic agents, have been used in clinical settings for a long time to suppress aberrant electrical activity by blocking the peak I_Na_. However, these drugs often come with a narrow therapeutic index and significant side effects. The increasing recognition of late I_Na_ as a pathological contributor in various cardiac disorders has led to the development of selective late I_Na_ inhibitors, which offer improved specificity and safety. Table 1 provides an overview of both classical and investigational agents with known or emerging activity against Nav1.5, excluding SGLT2 inhibitors, which are discussed in detail in Section 7.

**Table 1:** Provides an overview of both classical and investigational agents with known or emerging activity against Nav1.5, excluding SGLT2 inhibitors.

Drug name	Description	Year (FDA approval)	Effect on cardiac action potential duration	Cardiac safety screening outcome with regulatory/clinical justification	Mechanism of action (Nav1.5)	Figure
Quinidine [75]	Class I antiarrhythmics drug. But no longer in use due to side effects from March 2019	2010	Prolongs cardiac action potential duration (APD) (QTc)	Pro‐arrhythmic risk. Use restricted due to TdP risk, discontinued	Blocks peak I_Na_	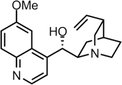
Procainamide [76]	Procainamide injection is used to treat irregular heartbeats and to slow an overactive heart	1950	Prolongs APD (QTc)	QTc prolongation, risk for TdP. Monitored for arrhythmia risk, labeled warning	Blocks peak I_Na_ and slow down the recovery after repolarization	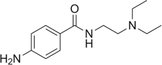
Disopyramide [77]	Class I antiarrhythmics drug. Mainly used for ventricular tachycardia/fibrillation	1977	Prolongs APD, strong QTc effect	Known pro‐arrhythmic effect. Strict indication, ECG required	Blocks peak I_Na_	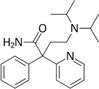
Lidocaine [78]	It is a local anesthetics and class IB antiarrhythmic drug	1999	Shortens APD (no QTc)	Minimal pro‐arrhythmic risk. Preferred for ventricular arrhythmias	Blocks peak I_Na_ by reducing its amplitude and shifting the voltage dependence of inactivation	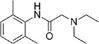	
Mexiletine [79]	Class IB antiarrhythmic drug with many side effects and less effectiveness	1998	Shortens APD	Minimal risk at therapeutic dose. Used for LQT3/chronic arrhythmia	Mainly, it decreases the peak I_Na_, while in some cases, it can increase peak I_Na_	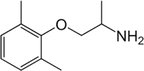	
Tocainide [77]	It is used to correct irregular heartbeats to a normal rhythm	1984	Shortens APD	Withdrawn for safety reasons (toxicity)	Blocks peak I_Na_	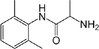	
Phenytoin [80]	Phenytoin is a class IB antiarrhythmic drug which rises the conduction in myocardial intersections, reduces the period of action potential in damaged cardiac tissue, and increases the threshold of ventricular fibrillation	1939	Shortens APD	Minimal arrhythmia risk. Used mainly as anticonvulsant	Blocks peak I_Na_	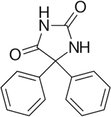	
Encainide [77]	Class Ic antiarrhythmic agent	1986	Minimal APD effect, slows conduction	Risk for arrhythmia, Withdrawn due to pro‐arrhythmic events	Blocks peak I_Na_	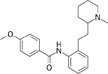	
Flecainide [81]	Treat abnormally fast heart rates. But has side effects like allergy	1985	Minimal APD effect, slows conduction	Pro‐arrhythmic risk in structural heart disease. Labeled for use only in non‐structural arrhythmia	Blocks peak I_Na_	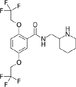	
Moricizine [82]	Class Ic antiarrhythmic drug. But withdrawn in 2007 for commercial reasons	1990	Minimally prolonged APD	Less pro‐arrhythmic than other Class IC. Use restricted, withdrawn in US	Blocks peak I_Na_ in atrial myocytes. It also also inhibited late I_Na_ under therapeutic concentrations	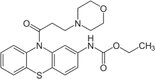	
Propafenone [83]	Class Ic antiarrhythmic drug	1989	Minimal APD effect, slows conduction	Pro‐arrhythmic in structural heart disease. Used chronically for AF in select patients	Blocks peak I_Na_ specially during open state	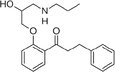	
KC12292 [84]	A thiadiazol derivative having antiischemic qualities is KC12292. By decreasing the sustained component (or slowly or no inactivating) of sodium current, this chemical also inhibits the inward peak I_Na_	Still in development phase	Data limited	Preclinical data only	Blocks peak I_Na._ It also blocks late I_Na_ in guinea‐pig atrial myocytes with an IC_50_ value of 9.6 × 10^−6^ *M*	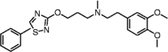	
Ranolazine [85]	It is used to treat chronic chest pain (angina)caused by a type of heart disease, coronary artery disease	2006	Minimal/shortens APD	Cardiac safety trials negative for QT effect. Labeled as antianginal, used off‐label	Blocks both peak and late I_Na_	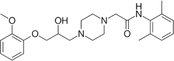	
F15845 Vacher [86]	A benzoxathiepine derivative drug that selectively blocks the sodium current to treat angina	Investigational stage, not approved by FDA yet	Minimal APD effect	Preclinical data only	reduced the persistent sodium current activated by veratridine (IC_50_ 1.58 × 10^−6^ mol·L^−1^)	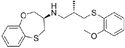	
RSD1235 (Vernakalant) [87]	Cardiome Pharma created Vernakalant, an antiarrhythmic medication designed to quickly induce sinus rhythm from atrial fibrillation	Vernakalant (Brinavess; Correvio Pharma) got FDA denied on 2008 and 2019 but in use in 41 countries including Canada, European nations, and a number of Asian countries like India, Bangladesh since 2010 Md [88]	No significant APD effect	Not approved by FDA, approved in Europe	Blocks peak I_Na_	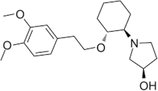	
AZD7009 [89, 90]	A mixed ion channel blocker, AZD7009 primarily affects the atrial tissue electrophysiologically by blocking potassium and sodium currents	It was discontinued from further development for non‐cardiovascular reasons. Vos [91]	Minimal APD effect	Preclinical data only	Blocks peak I_Na_	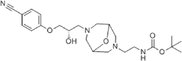	
Eleclazine (GS 6615) (compound 4 by Gilead Sciences) [92]	It is an investigational antiarrhythmic drug that selectively inhibits late sodium current (late I_Na_) in cardiac myocytes, aiming to reduce arrhythmogenic activity in conditions such as Long QT Syndrome type 3 and ventricular tachycardia	In 2015 orphan drug designation was assigned to the product by the FDA for the treatment of congenital long QT syndrome	Minimal APD effect	Preclinical data only	Blocks late I_Na_	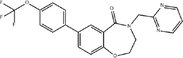	
Riluzole [93]	It is a drug used for the treatment of amyotrophic lateral sclerosis (ALS) that also functions as a sodium channel blocker with Ca^2+^‐activated K^+^ channel–enhancing properties, reduces intracellular Na^+^ accumulation by inhibiting the late Na^+^ current, suppresses Ca^2+^ leak, and may improve cardiac contractility and relaxation	1995	Minimal APD effect	Safety trials, no cardiac indication. Used for ALS, antiarrhythmic effects experimental	Blocks late I_Na_	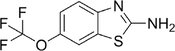	
Compound F15741 [94]	It is a selective, potent inhibitor of the late current mediated by the cardiac isoform of the sodium channel (Nav1.5)	Still in clinical trial not approved by FDA	Minimal APD effect	Preclinical data only	Blocks late I_Na_	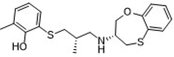	
Chinese Medicine Wenxin Keli (lauric acid) [95]	Lauric acid inside the Chinese Traditional Medicine Wenxin Keli is a key potent blocker for sodium channel Nav1.5 and prevents arrhythmia via inhibiting Late I_Na_	Approved by China Food and Drug Administration but not approved by FDA	Data limited	Traditional use in china only. Not FDA approved	Blocks late I_Na_	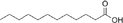	

In addition to traditional and investigational Nav1.5 modulators, a distinct class of compounds developed initially for metabolic disorders, SGLT2 inhibitors, has emerged as potential modulators of cardiac sodium currents, particularly with a reported preference for inhibiting late I_Na_ over peak I_Na_. However, their primary mechanism of cardio‐protection remains under investigation and likely involves multiple pathways beyond sodium channel modulation; their unique interaction with Nav1.5 warrants closer examination. In the following section, we analyze the electrophysiological and structural insights related to SGLT2 inhibitors, not to overstate their established therapeutic mechanisms, but to explore whether they might serve as a conceptual scaffold for the design of future late I_Na_ selective modulators.

## 7 Comparative Analysis of SGLT2 Inhibitors as Potential Modulator of Cardiac Sodium Currents

Several drugs, including ranolazine, eleclazine, and GS‐967, have shown that they can suppress late I_Na_ and reduce arrhythmias, supporting late I_Na_ as a useful therapeutic target [96]. Interestingly, common glucose‐lowering drugs like sodium–glucose cotransporter 2 inhibitors also influence late I_Na_, attracting significant interest.

Drugs like empagliflozin, dapagliflozin, and canagliflozin have been found to lower late I_Na_ in native heart cells and recombinant Nav1.5 systems, usually with little effect on peak I_Na_ under normal conditions. Notably, these effects appear more pronounced under pathological conditions, such as oxidative stress or heart failure models, where late I_Na_ is elevated [97]. However, these findings are context‐dependent, and the extent to which late I_Na_ modulation contributes to the cardioprotective effects of SGLT2 inhibitors remains incompletely resolved.

Not all drugs in this class behave similarly. For example, ertugliflozin shows little to no inhibition of late I_Na_ despite structural similarities, suggesting minor differences can impact interactions with Nav1.5 [19, 98]. On a molecular level, evidence suggests SGLT2 inhibitors may bind within the DIII–DIV region of Nav1.5. Residues like F1760 W1345, S1710, and S1458 are involved in drug binding, aligning with known antiarrhythmic sites [12, 99, 100]. Structural and computational studies indicate that dapagliflozin and canagliflozin bind similarly to empagliflozin, supporting a common interaction framework.

A very recent complex model proposes multi‐site or dual‐pocket binding, particularly for dapagliflozin, which can engage adjacent sites simultaneously, stabilizing the interaction and possibly affecting channel function [19]. Conversely, ertugliflozin's interaction involves an extra oxygen atom forming a hydrogen bond with residue Y1767, linked to pathological late I_Na_, though this is seen only in dual‐binding models [19, 101].

In addition, mechanisms beyond direct channel inhibition are suggested, such as dapagliflozin reducing oxidative CaMKII activation and electrical remodeling in atrial fibrillation models [102]. Some studies also propose that SGLT2 inhibitors increase Nav1.5 surface expression and help restore peak I_Na_ in heart failure, indicating broader effects. Overall, evidence points to SGLT2 inhibitors influencing late I_Na_ under certain conditions, but this varies depending on cell type, channel state, and structural factors. Importantly, late I_Na_ inhibition alone probably doesn't fully explain their heart benefits [103]. From a therapeutic view, these findings offer opportunities and challenges, while SGLT2 inhibitors are useful for studying late I_Na_, more research is needed to understand their mechanisms and develop more targeted, selective drugs. Figure 4 shows the structure of all of these drugs.

**Figure 4:**
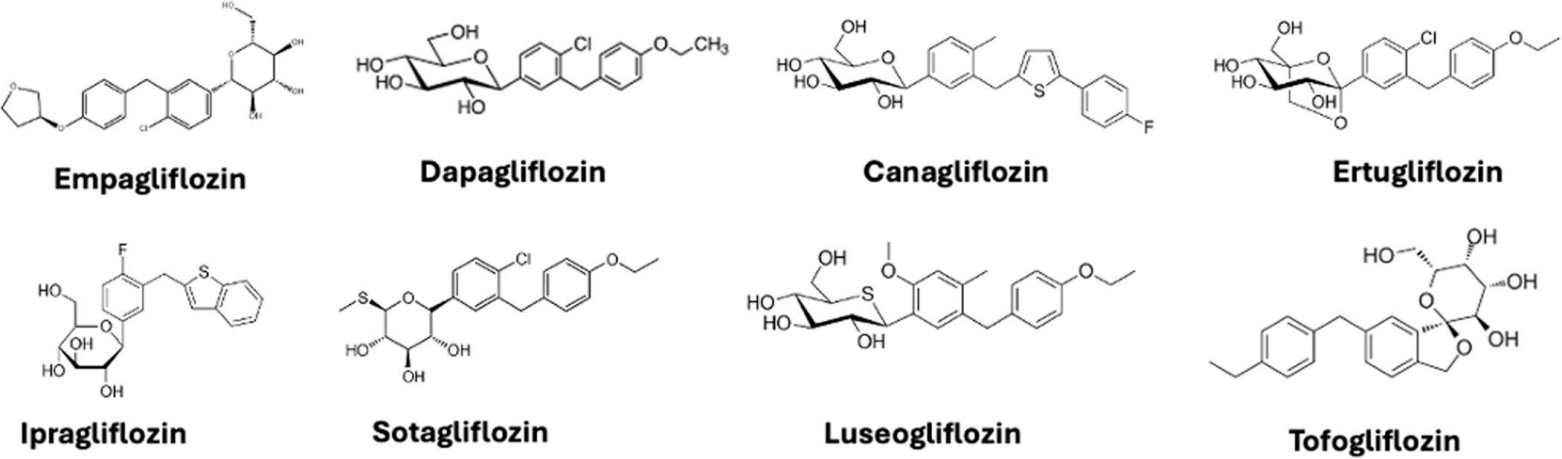
Different SGLT2is chemical structures.

## 8 Computational Approaches to Elucidate Nav1.5 Structure–Function Relationships and Guide Late I_Na_‐Selective Drug Discovery

In recent years, computational modeling has emerged as a crucial tool for investigating the structure–function relationships of VGSCs, including Nav1.5, and for guiding the design of selective modulators, particularly those targeting the late I_Na_. These models enable the simulation of channel conformational states, drug‐binding events, and mutational effects at both atomic and coarse‐grained (CG) resolutions. Although there are very few comprehensive in silico models of Nav1.5‐drug interactions currently available for late I_Na_ selective compounds, available methodologies offer a strong framework for filling this gap. In this section, we briefly review relevant all‐atomistic (AA), CG, and multiscale modeling strategies and highlight how they could be applied to advance structure‐based design of Nav1.5 inhibitors.

### 8.1 AA Molecular Modeling

AA molecular dynamics simulations provide the highest level of structural detail by explicitly representing every atom in the protein, membrane, solvent, and ligand. This enables precise investigation of intramolecular interactions, conformational changes, and ligand binding mechanisms at atomic resolution. Several studies have employed AA molecular dynamics simulations to explore the interactions between Nav1.5 and various small molecules, including SGLT2 inhibitors and established drugs such as ranolazine [12, 18, 22]. In silico experiments identified two preferred binding sites for empagliflozin within the Nav1.5 channel—specifically, the DIII–DIV (fDIII‐DIV) and DI–DIV (fDI‐DIV) fenestration regions—based on homology modeling, molecular docking, and molecular dynamics simulations. The strongest predicted binding affinity was observed at the fDIII‐DIV site, where empagliflozin interacts with key hydrophobic and aromatic residues such as F1760 and W1345, which are also involved in binding traditional sodium channel inhibitors like ranolazine and lidocaine [12]. Advances in homology modeling have facilitated detailed structural reconstructions of Nav1.5, including full‐length models based on cryo‐EM structures (PDB: 7DTC and 6LQA). In a recent study, an open‐state homology model of human Nav1.5 was built using the SWISS‐MODEL server and used to investigate the binding dynamics of late I_Na_‐modulating drugs [22, 104]. Missing intracellular loops, including regions with CaMKII phosphorylation sites, were modeled using the full‐length SCN5A sequence (Uniprot Q14524) as input. This refined model, which retained all four transmembrane domains (DI–DIV), was then used for in silico mutational analysis and docking of inhibitors such as empagliflozin and dapagliflozin, helping to identify critical binding residues, including F1760, W1345, and Y1767 [19]. These simulations offer a structural basis for understanding how different small molecules interact with Nav1.5 at specific fenestration sites and how subtle structural changes may influence the inhibition of late sodium current. In another recent complementary study, blind docking simulations were performed using the SeamDock web server with the cryo‐EM‐resolved rat Nav1.5 structure (PDB: 6UZ3), which shares 96% homology with human Nav1.5 [103]. Structural models of dapagliflozin and empagliflozin retrieved from DrugBank were energy‐minimized and docked across the full‐length channel using AutoDock4. The most favorable binding poses (ΔG = –7.41 kcal/mol for dapagliflozin and –7.85 kcal/mol for empagliflozin) were located within the DIII–DIV fenestration region of Nav1.5, aligning with findings from other independent modeling platforms. Notably, while both drugs occupied the same general region, empagliflozin was predicted to bind slightly deeper towards the pore. Key interacting residues included W1345, L1413, F1465 (S6 DIII), I1756, and F1760 (S6 DIV), highlighting a constellation of hydrophobic and hydrogen‐bond interactions that may stabilize the drug‐channel complex. These computational insights support the hypothesis that the DIII–DIV interface functions as a pharmacologically relevant hotspot for the selective modulation of Nav1.5 by SGLT2 inhibitors. Figure 5 shows the AA model of Nav1.5 bound to dapagliflozin, with the membrane and water.

**Figure 5:**
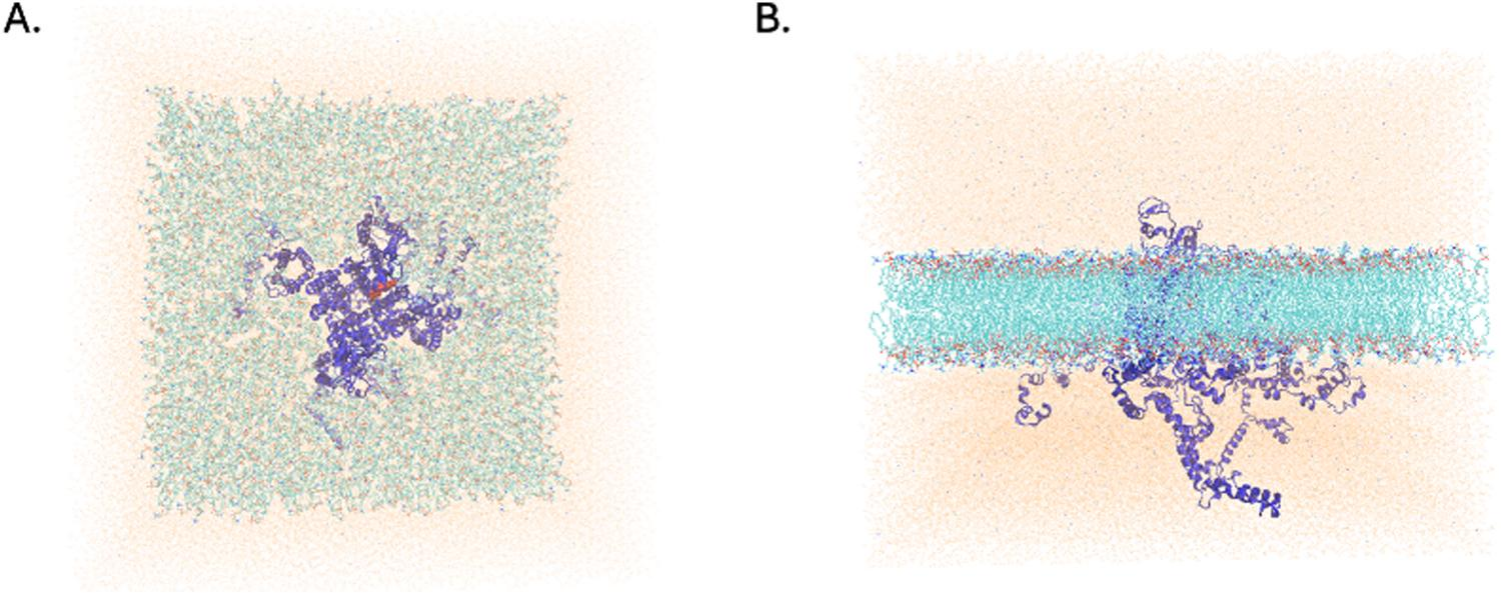
Full structure of Nav1.5 bound with dapagliflozin drug. (A) Top view‐ Nav1.5 in blue color, dapagliflozin in red, membrane in light blue or sky color, water in orange color. (B) Side view of the system in the same color pattern. [Color figure can be viewed at wileyonlinelibrary.com]

### 8.2 Multiscale Modeling and Artificial Intelligence (AI)‐Based Tools

Effectively exploring complex biological systems, such as Nav1.5, necessitates the utilization of multiscale modeling, especially when aiming to simulate processes occurring over microsecond timescales. Despite their detailed nature, AA simulations are often prohibitively time‐consuming and computationally intensive, rendering them unsuitable for extensive simulations or intricate systems. Researchers can achieve an optimal balance and efficacy in simulations by focusing on atomic‐level detail exclusively on essential components, such as the protein and ligand, while simplifying less critical elements, including the surrounding membrane and water, into CG levels. In CG models, multiple atoms are aggregated into single interaction sites, commonly known as “beads,” thereby reducing the total number of particles within the system. For example, in widely used CG water models like MARTINI, four AA water molecules are represented by a single CG bead. Multiscale modeling, which integrates models across different levels of complexity, provides a robust methodology for capturing vital chemical interactions while conserving computational resources. Recent advancements in this domain have enhanced the capacity to study large, dynamic membrane proteins such as Nav1.5 with increased efficiency and resolution. Force fields such as SIRAH and PACEm facilitate hybrid frameworks that combine atomistic detail—particularly at critical regions like ligand‐binding sites or gating interfaces—with CG representations of the surrounding membrane and solvent [105, 106, 107]. While SIRAH is noted for its versatility across major simulation platforms, including AMBER, GROMACS, and NAMD, its compatibility with fully atomistic Nav1.5 proteins is still under development. Conversely, PACEm, an extension of the PACE force field, enables coupling between united‐atom and CG models and has been successfully employed to study complex ion channels such as Piezo1 [107]. Although PACEm demonstrates considerable promise for capturing both subtle and significant motions in Nav1.5, current limitations involve the absence of validated protocols for ligand incorporation. Overall, these multiscale approaches represent a promising avenue for simulating Nav1.5 dynamics with reduced computational expenditure, especially when investigating conformational changes and potential allosteric drug effects. A particularly challenging region within Nav1.5 is the DIII–DIV linker, a crucial structural element involved in fast inactivation and conformational switching. Capturing its large‐scale, slow‐timescale dynamics remains impractical with traditional AA simulations due to computational constraints. Although no published studies have yet demonstrated this approach for Nav1.5. Although a recent study shows prospective direction involves deriving CG models from existing AA structures to facilitate extended CG simulations, subsequently backmapping the CG models into AA resolution for detailed analysis [19]. This workflow may provide a viable strategy to overcome current limitations and enable the exploration of long‐timescale conformational transitions within critical regulatory regions of Nav1.5.

In addition to physics‐based simulations, emerging AI and deep learning methods are increasingly supplementing traditional models in ion channel research. Structure‐based generative models and AI‐driven drug design platforms, such as TamGen, REINVENT 4 and AI‐MedCraft, can quickly explore chemical space and suggest novel scaffolds with predicted binding affinities for targets like Nav1.5 [108, 109]. Nonetheless, their use for Nav1.5 and specifically for late I_Na_ selective modulation remains limited. A major challenge is accurately translating predicted binding interactions into functional effects, such as changes in inactivation kinetics. Therefore, AI approaches are best seen as complementary to molecular simulations, needing to be integrated with mechanistic and electrophysiological validation to develop reliable late I_Na_ modulators.

## 9 Lessons Learned From the Development of Nav Channel Inhibitors: Implications for Nav1.5

The development of Nav1.5 selective inhibitors can benefit significantly from the insights gained through the design and optimization of other Nav channel modulators, particularly Nav1.7 and Nav1.8 inhibitors. One notable example is the discovery of DS‐1971a, a potent and selective Nav1.7 blocker by Japanese scientists [110, 111]. Structural optimization strategies, such as modifying the phenyl ring and substituting a 4‐pyridyl moiety, improved potency, reduced CYP3A4 inhibition, and lowered the risk of idiosyncratic toxicity. DS‐1971a demonstrated favorable safety, tolerability, and pharmacological profiles in both preclinical and early clinical studies for neuropathic pain, making it a promising template for selective Nav channel inhibition.

Furthermore, a recent study employed in silico screening methods to identify high‐affinity ligands exhibiting up to 40‐fold selectivity for Nav1.7, thereby demonstrating the utility of computational techniques in achieving specificity among closely related isoforms [112]. Although clinical outcomes for Nav1.7 inhibitors have been inconsistent, these investigations emphasize the significance of precise design, ADME profiling, and innovative delivery systems in the development of modulators. Emerging strategies such as CRISPR/Cas9‐mediated downregulation of SCN9A and gene therapy platforms like SMARTmid (PepVax Inc) [113] exemplify alternative approaches with long‐term potential.

The recent FDA approval in January 2025 of suzetrigine, a Nav1.8 inhibitor, marks a notable milestone in the management of chronic pain and provides a concrete example of successful drug development. The progression of suzetrigine through clinical trials accentuates the significance of selectivity, safety, and efficacy in translating Nav modulators into approved therapeutics. Collectively, these experiences indicate that meticulous isoform targeting, advanced in silico modeling, and the integration of genetic and pharmacological strategies will be vital for future development of Nav1.5‐targeted drugs, especially in differentiating between peak and late I_Na_ modulation.

## 10 Clinical Translation of Nav1.5 Modulation: From Bench to Bedside

In clinical translation, the term “bench to bedside” refers to the process of translating scientific research findings from the bench (research laboratory) to patient treatment at the bedside. The transition from basic research on Nav1.5 to clinical application involves directing the therapeutic complexity of modulating the late I_Na_. Although late I_Na_ inhibition has shown promise in reducing arrhythmic risk in conditions such as heart failure, clinical translation has been challenging due to complicated pharmacological and physiological factors.

One prominent example is Gilead Sciences’ late I_Na_ inhibitor program, which faced setbacks despite promising preclinical work [114]. Compounds such as ranolazine demonstrated antiarrhythmic effects but also showed off‐target interactions with other cardiac currents, particularly potassium current (I_Kr_), complicating their safety profile and clinical efficacy [115, 116]. Non‐selective sodium channel blockade can lead to QT prolongation and an increased risk of proarrhythmia, especially in patients with underlying cardiac vulnerabilities [117].

Moreover, late I_Na_ may serve compensatory roles in certain pathophysiological states. For example, in heart failure, increased late I_Na_ may help maintain action potential duration and calcium handling [118]. Thus, unselective inhibition could disrupt protective mechanisms, underscoring the importance of precise patient selection and dosing strategies.

Past clinical trial designs often failed to capture these notes, emphasizing the need for more targeted trials with carefully chosen endpoints and subpopulations. Furthermore, despite the development of more selective inhibitors like compound 2(GS‐458967) by Gilead Sciences, clinical progress remains limited due to pharmacokinetic and safety challenges [119]. A similar issue happened with them again with compound 3 (GS‐462808) as the development was not pursued due to liver lesions in 7‐day rat toxicology studies [120].

These observations do not invalidate late I_Na_ as a therapeutic target, but rather underscore the need for greater selectivity, improved mechanistic understanding, and refined clinical trial design. Future inhibitor development must aim to dissociate pathological late I_Na_ from physiological sodium current functions, ensuring both safety and efficacy.

In conclusion, the failure of earlier attempts to synthesize effective late I_Na_ inhibitors can be attributed to several factors, including non‐selective inhibition of multiple ion currents, the physiological role of late I_Na_ under specific conditions, and pharmacokinetic limitations. Moving forward, the development of more selective compounds that target late I_Na_ without disrupting other essential cardiac currents is crucial. Computational modeling—including atomistic and multiscale simulations—offers a powerful strategy to guide the rational design of such selective inhibitors and to predict their effects in silico before advancing to clinical testing.

## 11 Conclusion

This review discusses late sodium current (late I_Na_) as a distinct and therapeutically important aspect of Nav1.5 function. While Nav1.5 is key in heart electrophysiology, only some of its pathological effects, especially in conditions like LQT3, are directly caused by increased late I_Na_. This distinction is vital because it helps identify when targeting late I_Na_ is most effective. Advances in structural and mechanistic research have shown that late I_Na_ results from complex changes in channel inactivation, gating, and post‐translational processes. These findings provide a basis for designing targeted therapies but also present a challenge: how to modulate late I_Na_ selectively without affecting the normal peak I_Na_. SGLT2 inhibitors are a notable, yet not fully understood, example of drugs that can influence late I_Na_ under certain conditions. Their effects vary within the drug class and appear to depend on context, indicating that inhibiting late I_Na_ alone does not completely explain their heart‐protective effects. More research into their mechanisms and structure–activity relationships is needed. Moving forward, combining structural biology, electrophysiology, and computational modeling will be crucial for progress. New computational methods, as well as AI‐based methods, could help identify residue interactions and assist in designing more selective drugs that target inactivation‐specific pathways. In conclusion, late I_Na_ is a promising but complex therapeutic target. Success will depend on identifying the disease states where late I_Na_ is a key factor, developing precise modulation strategies, and turning mechanistic insights into effective clinical treatments.

## Data Availability

No new data were created or analyzed in this study. Data sharing is not applicable to this article.
